# Aged cells in human skeletal muscle after resistance exercise

**DOI:** 10.18632/aging.101472

**Published:** 2018-06-27

**Authors:** Chi Yang, Ying Jiao, Bing Wei, Zeyi Yang, Jin-Fu Wu, Jorgen Jensen, Wei-Horng Jean, Chih-Yang Huang, Chia-Hua Kuo

**Affiliations:** 1 Laboratory of Exercise Biochemistry, University of Taipei, Taipei, Taiwan; 2 Competitor Institute of Sports Nutrition, Beijing, China; 3 Norwegian School of Sport Science, Oslo, Norway; 4 Department of Anesthesiology, Far East Memorial Hospital, New Taipei, Taiwan; 5 Graduate Institute of Basic Medicine, China Medical University, Taichung, Taiwan; 6 Department of Biological Science and Technology, Asia University, Taichung, Taiwan; * Equal contribution

**Keywords:** anti-aging, inflammation, p16Ink4a, macrophage, whey protein

## Abstract

It remains unclear how exercise, as an entropic event, brings benefit against human aging. Here we examined longitudinal changes of p16^Ink4a+^ senescent cells in skeletal muscle of young men (aged 22.5±1.7 y) before and after resistance exercise (0 h and 48 h) with multiple biopsies at two different protein availabilities: low protein (14%) and isocaloric high protein (44%) supplemented conditions. Immunohistochemistry analysis of muscle cross-sections using p16^Ink4a^ and CD34 antibodies confirmed that the detected senescent cells were endothelial progenitor cells. Leukocyte infiltration into skeletal muscle increased during resistance exercise. The senescent cells in muscle decreased (-48%, P < 0.01) after exercise for 48 h. Low protein supplementation resulted in greater infiltrations of both CD68^+^ phagocytic macrophage and leukocyte, further decreased p16^Ink4a+^ senescent cells (-73%, P < 0.001), and delayed increases in regenerative CD163^+^ macrophage in skeletal muscle, compared with high protein supplemented condition. Significant gain in muscle mass after 12 weeks of training occurred only under high protein supplemented condition. Conclusion: Rapid senescent cell clearance of human skeletal muscle during resistance exercise seems to associate with enhanced *in situ* phagocytosis. High protein availability accelerates resolution of muscle inflammation and promotes muscle increment after training.

## Introduction

Most of the cells in the human body are continuously aging, dying and regenerating to gradually evolve a fairly stable size of multicellular system with a wide range of cell ages [1]. Skeletal muscle is the largest tissue of the human body, in which cell lifespan varies considerably among different cell types. For example, myofibers are long-lived, whereas endothelial cells in capillary surrounding myofibers age rapidly with a short half-life around 2 weeks [2]. Selective elimination of senescence cells in skeletal muscle and other tissues has been shown to increase lifespan in mice [3], suggesting a promising approach for anti-aging intervention. The protein p16^Ink4a^, a cyclin-dependent kinase inhibitor CDKN2A, is a widely used senescence marker expressed specifically in aged cells [4–6]. However, p16^Ink4a+^ senescence cells in human skeletal muscle are rarely studied. It is currently unclear whether senescent cells are accumulated in human skeletal muscle at young age and whether exercise has significant influence on its number.

Senescent cells can be selectively recognized and rapidly cleared by phagocytic macrophage [7]. One way to direct macrophages into skeletal muscle is resistance exercise [8]. After weight loading, phagocytic macrophage (M1, CD68^+^) infiltrated into damaged sites, followed by protracted presences of regenerative macrophage (M2, CD163^+^) [9,10]. The cell turnover process instantly demands nitrogen sources from amino acids or proteins for nucleotide synthesis and DNA replication [11]. A delayed protein supplementation after resistance training can significantly undermine muscle hypertrophy [12,13], suggesting a far-reaching impact of protein availability in time around exercise challenge on long-term muscle adaptation. It remains uncertain whether protein availability influences macrophage presences and senescent cell clearance in exercising skeletal muscle.

In this study, senescent cell distribution and quantity in vastus lateralis muscle were examined in young human adults after a single bout of resistance exercise. To determine the effects of dietary protein availability around exercise on senescent cell quantity and macrophage infiltration of skeletal muscle, two isocaloric protein supplements (14% and 44% in calorie) were ingested before and immediately after an acute bout of resistance exercise, in a counter-balanced crossover fashion. An additional parallel trial was conducted to compare the outcome of muscle mass increment under the same dietary conditions after 12 weeks of resistance training.

## RESULTS

**Figure 1:**
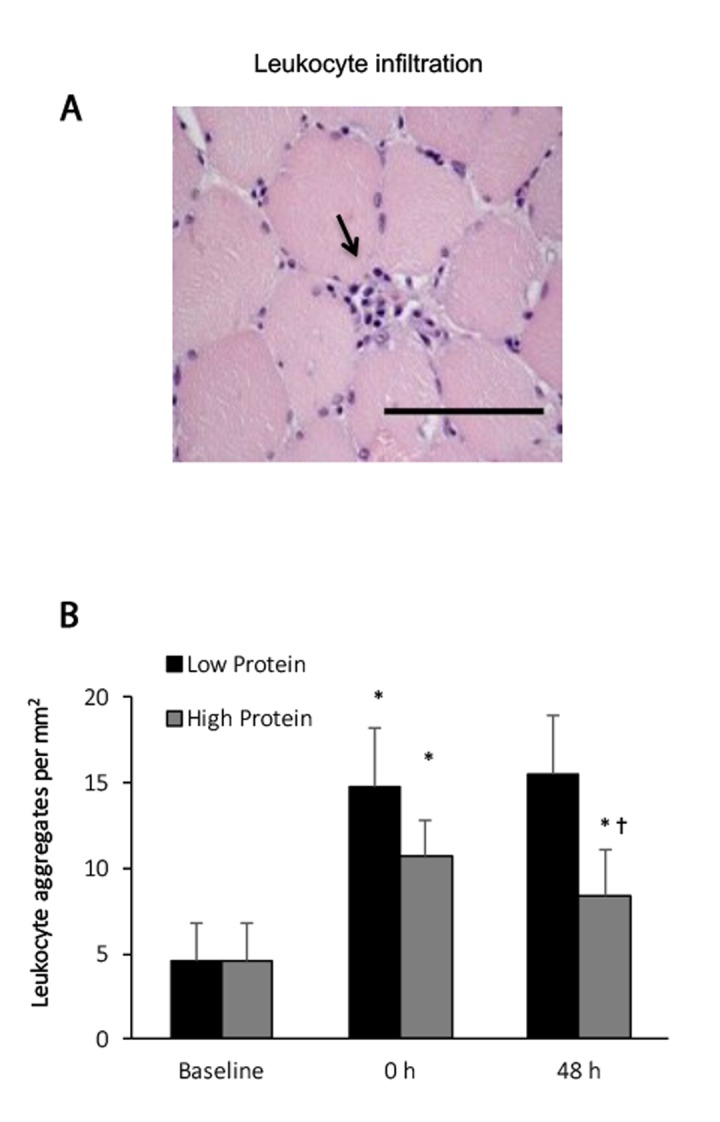
**Leukocyte infiltration in human skeletal muscle after resistance exercise.** (**A**) Representative hematoxylin and eosin staining of a muscle cross-section (leukocytes infiltration indicated by an arrow). (**B**) Resistance exercise immediately increased leukocyte infiltration into skeletal muscle. High protein supplementation before and after resistance exercise attenuated exercise-induced leukocyte infiltration. * Significant difference against Baseline, P < 0.05; † Significant difference against Low Protein, P < 0.05. Low protein: 14% protein; High protein: 44% protein in weight.

Blood leukocyte concentrations decreased moderately from 6233 ± 629 to 5600 ± 572 count per microliter (-14% below baseline), 48 h after an acute bout of resistance exercise (main effect, P < 0.05). No significant interactive effect of time and supplementation was found. Leukocyte infiltrations in exercised muscle increased significantly by 82% and 230% above baseline during the high protein and low protein supplemented trials, respectively, 48 h after the challenge (Figure 1). No significant interactive effect of time and supplementation was observed. Exercised muscle in the low protein trial shows greater increases in leukocyte than that in the high protein trial (P < 0.05).

**Figure 2:**
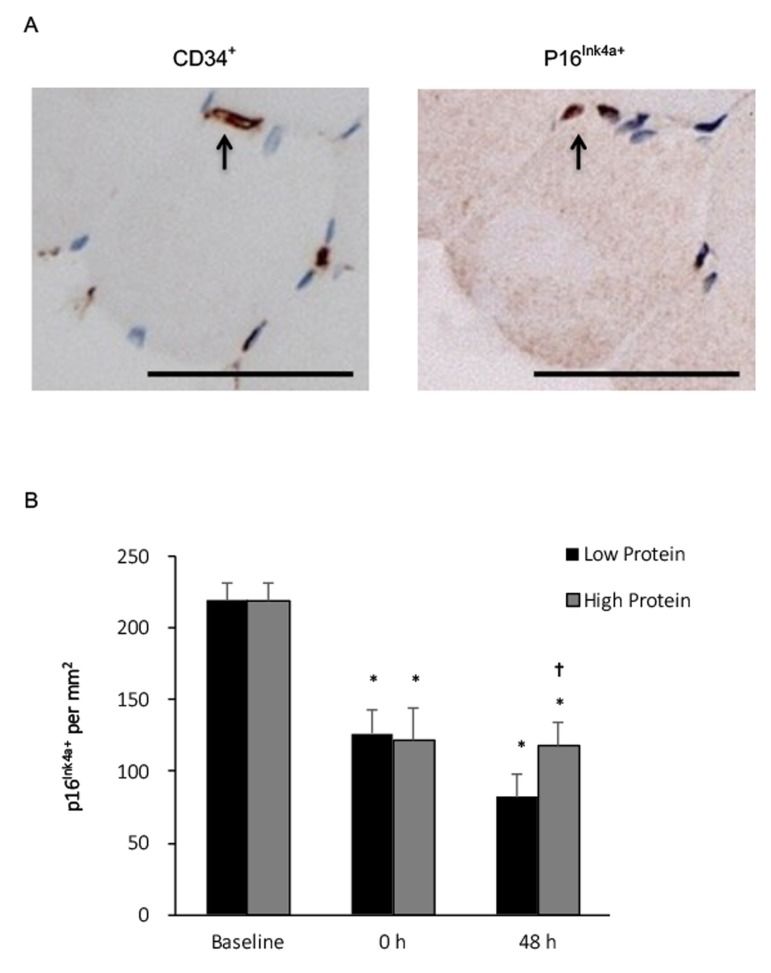
**Senescent endothelial progenitor cells (p16^Ink4a+^/CD34^+^) in human skeletal muscle after resistance exercise.** (**A**) Representative immunohistochemical co-staining of muscle cross-sections (senescent cells indicated by arrows). Scale bar 550 mm. (**B**) Senescent endothelial progenitor cells decreased in human skeletal muscle after a single bout of resistance exercise, and to a greater extends under low protein supplemented condition. * Significant difference against Baseline, P < 0.05; † Significant difference against Low Protein, P < 0.05. Low protein: 14% protein; High protein: 44% protein in weight.

No p16^Ink4a+^ myofibers of the young men was detected. Co-localization of p16^Ink4a+^ and CD34^+^ signals on muscle cross-section at baseline reveals that the detected senescent cells in vastus lateralis muscle of young men were endothelial progenitor cells (Figure 2, upper panel). Before exercise, more than 40% of these endothelial progenitor cells (CD34^+^) displays a various degree of p16^Ink4a+^ signals around muscle fibers of the biopsied vastus lateralis muscle. Nearly half of p16^Ink4a^ positive cells were eliminated immediately after exercise for both low and high protein supplementation trials (P < 0.01) without a significant difference between trials. A slightly greater decrease in p16^Ink4a+^ cells was observed with the low protein supplemented condition 48 h after exercise (-73% vs. baseline, P < 0.001) compared with the high protein supplemented condition (Figure 2, lower panel). No significant interactive effect of time and supplementation was found.

**Figure 3:**
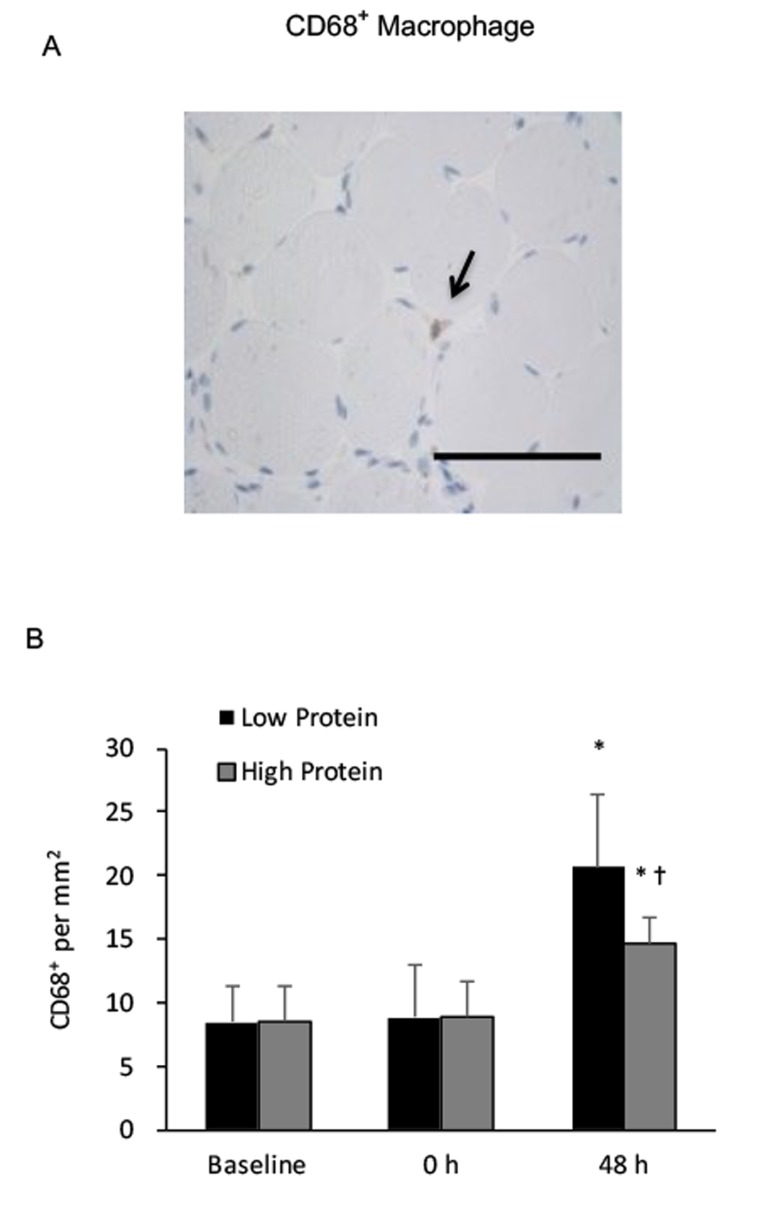
**Phagocytic macrophage (CD68^+^) in human skeletal muscle after resistance exercise.** (**A**) Representative immunohistochemical staining of a muscle cross-section (CD68^+^ macrophage indicated by an arrow). Scale bar 550 mm. (**B**) Low protein supplementation before and after resistance exercise enhanced CD68^+^ macrophage infiltration in skeletal muscle above High protein trial. * Significant difference against Baseline, P < 0.05; † Significant difference against Low Protein, P < 0.05. Low protein: 14% protein; High protein: 44% protein in weight.

**Figure 4:**
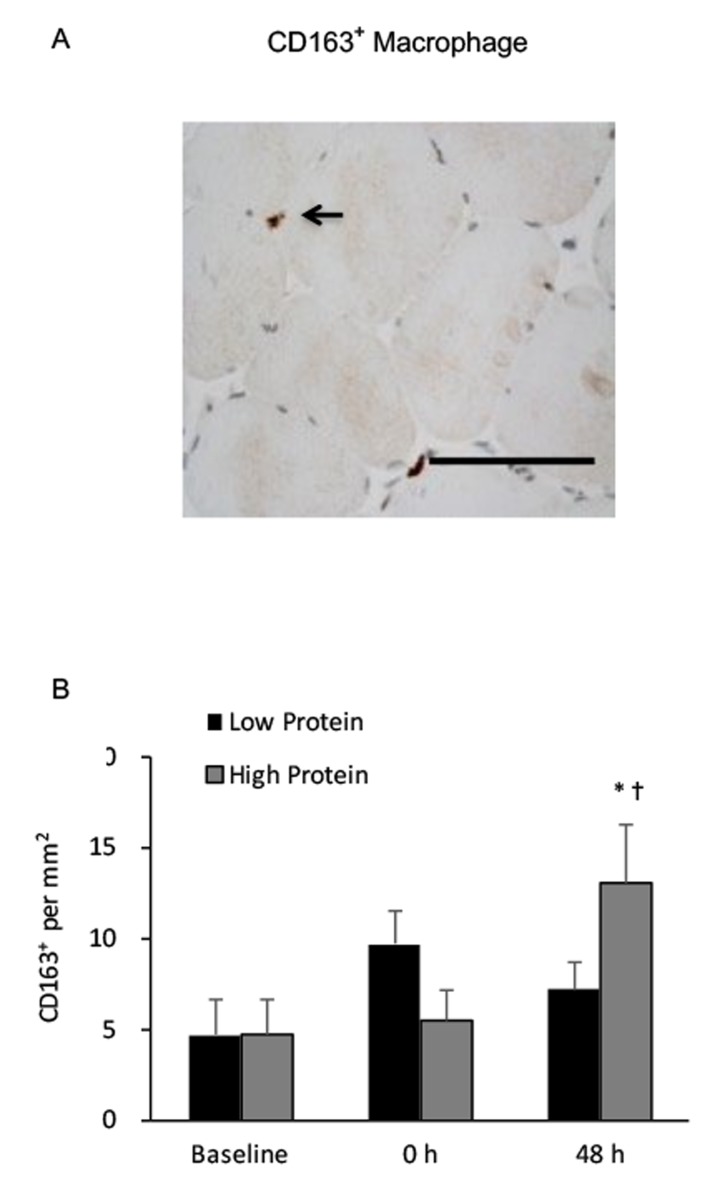
**Regenerative macrophage (CD163^+^) in human skeletal muscle after resistance exercise.** (**A**) Representative immunohistochemical staining of a muscle cross-section (CD163^+^ macrophage indicated by an arrow). Scale bar 550 mm. (**B**) High protein supplementation before and after resistance exercise increased CD163^+^ macrophage presence in human skeletal muscle 48 h after exercise. * Significant difference against Baseline, P < 0.05; † Significant difference against Low Protein, P < 0.05. Low protein: 14% protein; High protein: 44% protein in weight.

CD68^+^ macrophage infiltration in muscle increased by 150% and 80% above baseline during the low and high protein trials, respectively (main effect of training, P < 0.05) (Figure 3). No significant interactive effect of time and supplementation was found. Increased CD68^+^ macrophage infiltration in muscle after exercise was greater under the low protein supplemented condition than the high protein supplemented condition (p < 0.05). CD163^+^ macrophage of vastus lateralis muscle during the high protein trial significantly elevated by 180% above baseline (P < 0.01) (Figure 4), whereas no significant increase in CD163^+^ macrophage was observed during the low protein trial, 48 h after exercise. Significant interactive effect of time and supplementation was detected (P < 0.05).

**Figure 5:**
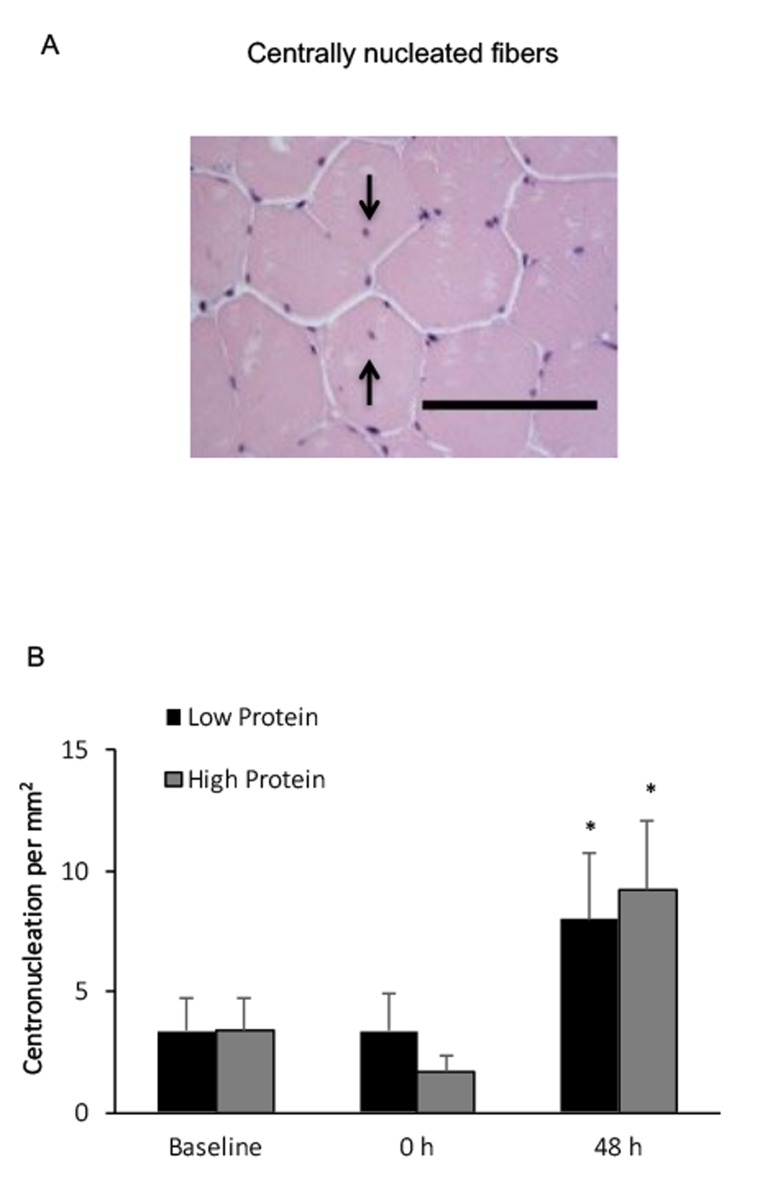
**Centrally nucleated fibers in human skeletal muscle after resistance exercise.** (**A**) Representative hematoxylin and eosin staining of a muscle cross-section (centrally nucleated fibers indicated by arrows). Scale bar 550 mm. (**B**) (**††**) No difference between Low and High protein trials was found. * Significant difference against Baseline, P < 0.05. Low protein: 14% protein; High protein: 44% protein in weight.

Centrally nucleated muscle fibers in the high and low protein trials significantly increased by 170% and 130%, respectively above baseline (Figure 5), 48 h after exercise. No significant difference between both trials was observed.

For the second study, muscle mass increased by ~ 1 kg after the 12-week resistance training in the high protein trial (Table 1), whereas the muscle mass change in the low protein trial was not reached statistical significance. Interactive effect of time and supplementation was not reached statistical significance.

**Table 1:** Muscle mass increment of young adults after 12 weeks of resistance training.

	Low Protein (14%)			High Protein (44%)		
Unit: Kg	Pre	Post	Change	Pre	Post	Change
Muscle mass	54.8±1.4	55.1±1.5	+0.40	56.6±1.3	57.7±1.1	+1.08 **
Fat mass	12.5±1.1	12.9±1.1	+0.38	14.3±1.6	14.7±1.7	+0.41
Body weight	70.3±2.1	71.1±2.2	+0.79	74.1±2.9	75.6±2.6	+1.49

Significantly muscle mass increment observed only under high protein supplemented condition. Muscle mass (bone-free lean mass) was measured by dual-energy X-ray absorptiometry. ** Significant difference against Pre, P < 0.01. Low protein: 14% protein; High protein: 44% protein in weight.

## DISCUSSION

### Major findings

In the study, we have identified the location of p16^Ink4a+^ senescent cells in human skeletal muscle, and measured the impact of resistance exercise on the quantity of p16^Ink4a+^ senescent cells under two different protein supplemented conditions. The main findings of the study are as follows: 1) No senescent myofibers are detected in the skeletal muscle of young men aged between 20-25 y; 2) Most of the senescent cells found around muscle fibers are endothelial progenitor cells; 3) A single bout of resistance exercise reduces the senescent endothelial progenitor cells significantly in challenged muscle and maintains at low levels for 48 h; 4) Resistance exercise with low protein availability is associated with greater increases in macrophage infiltration and further depletion of senescent endothelial progenitor cells in muscle tissue during recovery, but prevents muscle hypertrophy for a long term. Taken together, these data suggest that senescence cell clearance and muscle mass increment are associated with the magnitude of muscle inflammation after resistance exercise, which can be influenced by protein supplementation around exercise.

It is not surprising that more than 40% of CD34^+^ endothelial progenitor cells in skeletal muscle of healthy young men showed various degrees of P16^Ink4a+^ signals. The average lifespan of endothelial cells in cardiac muscle of animals lasts only ~2 weeks [2], suggesting that this cell type ages rapidly compared with myofibers in muscle tissue [1]. Endothelial cells in skeletal muscle may be viewed as a collection of fit and unfit cell populations, based on their capability to survive against physical challenges. Resistance exercise may weakens those unfit cells, leading to presentations of danger-associated molecular pattern to attract immune attack from phagocytic macrophage [14]. The selective scavenging mechanism by innate immunity may help to reinforce a multicellular system by recovering exercise muscle tissue with younger and healthier cell population for better survival against next similar challenges. Eliminating p16^Ink4a+^ senescent cells is known to significantly increases lifespan in mice [3]. Given the fact that resistance exercise increases muscle damage, the present results on senescent cell elimination provide an explanation on how a destructive challenge may bring benefit for human survival [15].

Resistance exercise immediately increased presences of leukocyte and phagocytic macrophage in challenged skeletal muscle [8]. The rapid decline of senescent endothelial progenitor cells after exercise may have been associated with the combined results of increased phagocytosis [7] and homing of new circulating endothelial progenitor cells (or termed blood outgrowth endothelial cells from bone marrow) in capillaries of challenged muscle [16,17]. Macrophage activation is a fast acting mechanism to clear the senescent cells in muscle tissue [10,18]. Selective elimination of human senescent cells by phagocytic macrophage has been reported [7], which can occur at an extremely fast pace. Our observation on different magnitudes of p16^Ink4a+^ senescent cell clearance in muscle tissue after resistance training under low and high protein trials fits well with the difference in the amount of CD68^+^ macrophage infiltration within tissue. An acute episode of tissue ischemia during eccentric muscle contractions may increase homing of circulating endothelial progenitor cells to challenged sites [16].

The current result suggests that low protein availability delays resolution of muscle inflammation and decreases muscle mass increment after resistance training. Inflammation is essential for muscle regeneration induced by weight loading [9,10]. During the 48-h recovery after resistance exercise, high protein supplementation attenuates increases in CD68^+^ macrophage infiltration and promotes earlier presence of CD163^+^ macrophage in the exercised skeletal muscle 48 h after workout, compared with low protein supplemented condition. The surge of regenerative CD163^+^ macrophage in challenged muscle occurs nearing completion of the phagocytic phase of inflammation [9]. Therefore, the result of the study suggests that increasing protein availability accelerates completion of the inflammation program in exercised skeletal muscle for recovery. Protein and amino acids are primary nitrogen source of nucleotide synthesis for DNA replication during cell proliferation [19], which is essential for regenerating endothelial cell during post-exercise recovery. Amino acids have been shown to promote endothelial cell proliferation in vitro [20]. The rate of endothelial cell proliferation in capillaries can occur at a very fast rate with a doubling rate around a day [21]. Prolonged CD68^+^ macrophage infiltration with low protein availability may cause more phagocytosis in challenged tissue to extract endogenous nitrogen source from existing muscle tissue in response to the increasing demand on DNA synthesis during regeneration. This may explain the results on insignificant muscle mass increment under low protein supplementation following a 12-week of resistance training. It is not new that delayed protein supplementation after resistance exercise can have a negative effect on muscle hypertrophy in a long term [12,13]. Therefore, the present data on more CD68^+^ macrophage and less CD163^+^ macrophage in human skeletal muscle 48 h after resistance exercise may be an indicative of delayed resolution of muscle inflammation due to low protein availability.

The major limitation of the study is that only a single type of training has been used to investigate the senescent cell clearance effect and the data should not be generalized to other exercises. Additional studies on exercise regimen that employs more concentric muscle contractions, such as swimming, cycling, and aerobic exercise, are required.

## CONCLUSION

Phagocytosis by macrophage is a known mechanism to selectively eliminate senescent cells in the human body. A single bout of resistance exercise increases infiltration of phagocytic macrophage into skeletal muscle and eliminates significant amount of senescent cells *in situ*. The results of the study provide possible explanations on how exercise as an entropic event brings benefit of anti-aging outcome observed in the past [15]. Furthermore, high protein availability may accelerate resolution of muscle inflammation and thus promotes muscle increment induced by resistance training.

## MATERIALS AND METHODS

### Ethical approval

Institutional Review Board at University of Taipei approved the study protocols (Approved number IRB-2016-014). The purpose, experimental procedure, and potential risks of participation were explained to all participants. This study was conducted in accordance to the principles set by the Declaration of Helsinki. Written informed consent was received before the study.

### Participants and study design

All participants were asked not to join in any resistance exercise and not to take special nutritional supplements since one month before and during the studies. Exclusion criteria were sedentary, cardiovascular, musculoskeletal, or inflammatory conditions (severe allergy, juvenile rheumatoid arthritis, dermatomyositis, or systemic lupus erythematosus).

Two studies were conducted with separate cohorts at similar age. Characteristics of participants of both studies are shown in Table 2.

**Table 2:** Baseline characteristics of participants. Low protein: 14% protein; High protein: 44% protein in weight.

Study 1	Crossover design (N = 12)	
Age (y)	22.5±1.7	
Weight (kg)	68.8±12.1	
Height (cm)	171.1±6.9	
Years engaged in sports	> 3 y	
Study 2	Parallel design	
Group	High Protein (N = 13)	Low Protein (N = 13)
Age (y)	21.4±0.2	21.4±0.2
Weight (kg)	74.1±2.9	70.3±2.1
Height (cm)	174.6±1.7	172±1.5
Years engaged in sports	> 3 y	> 3 y

Study 1: To avoid potential musculoskeletal injury caused by resistance exercise, inactive individual were precluded. There were 12 college students (non-athletes) who have engaged sports activity at least twice a week in the past 1-month before the study voluntarily participated in randomized counter-balanced crossover trials. The effects of an acute bout of resistance exercise on senescent cell number and macrophage infiltration in human skeletal muscle were examined under high protein supplementation and another occasion under low protein supplementation, separated by 3 weeks. The washout time was based on the time required for recoveries of muscle soreness and strength from resistance exercise documented elsewhere [8]. All participants were randomized into the two dietary conditions to compare themselves in a counter-balanced fashion. For each trial, half participants ingested low protein (or high protein) supplement before and immediately after the workout. On another occasion, participants ingested an isocaloric high protein (or low protein) supplement before and after the workout. Each trial involved an acute session of resistance exercise. Multiple muscle biopsies on leg muscle were conducted to assess the longitudinal changes of senescent cell number and macrophage infiltration in muscle tissues before and after (0 h and 48 h) exercise. The only difference between trials was the content of the supplements. Two participants dropped out due to time conflict. They were then excluded form pair-comparison between two supplemented conditions.

Study 2: To avoid potential musculoskeletal injury caused by weight training, 26 college students with experience of sports training for more than 3 years before the study were recruited and randomized into the high protein and low protein groups to evaluate the outcome of muscle mass increment (bone-free lean mass) by dual-energy X-ray absorptiometry (DEXA) after 12 weeks of resistance exercise. Participants were randomized into low and high protein supplemented groups and were instructed to consume the same supplement before and immediately after workout as the first study during a 12-week training program while maintaining their habitual daily supplementation.

### Protein supplements

For both studies, participants were asked to consume drinks before (7.2 kcal per kg) and after exercise (7.2 kcal per kg) made by low and high protein supplement powder (Beijing Competitor, Beijing, PROC). Whey protein isolate is the protein component. The high protein supplement composed of 44% protein, 54% carbohydrate, and 2% fat in weight (or 36% protein, 58% carbohydrate, and 6% fat in calories). The low protein supplement composed of 14% protein, 73% carbohydrate, and 13% fat in weight (or 12% protein, 63% carbohydrate, and 25% fat in calories). The low and high protein supplements were indistinguishable in appearance. Dietary control started 12 h before resistance exercise by receiving a regular meal. After a 10-h overnight fast, participants reported to the laboratory 3 h before each trial in the morning without breakfast before resistance exercise.

### Resistance exercise

A specialist supervised all training sessions to ensure the correct techniques and monitored the appropriate amount of exercises and rest intervals. One repetition maximum (1RM) testing was determined according to the a principle as previously described [22]. Before testing, each participant completed two warm-up sets consisting of 2-3 repetitions at ~40-60% of perceived maximum. The weight was increased incrementally by 14-18 kg with 4 min of rest between each set. Each participant performed up to 5 subsequent trials to determine 1RM. During the squat exercise, participants placed a safety squat bar across their shoulders and gradually descended until hip joint reached the same level as the knee. Participants then ascended to a complete knee extension. An additional chance will be given when participants could not complete the repetition or maintain proper range of motion. If they were still unable to complete the exercise correctly, the last completed weight was set as the 1RM.

For Study 1, each participant familiarized with the weight training facility by performing light squats before experiment. The resistance was set at 70% of each individual's 1RM. Each session was comprised of 6 sets of 12 repetitions of squat exercise under supervision of a training specialist to endure consistency of the practices.

For Study 2, a 12-week training program began after baseline assessments. The resistance was set at 70% of each individual's 1RM for both the high protein and low protein groups. Each session consisted 3 sets of 12 repetition (70% of 1RM) of exercises: bench press, front squat, back squat, bicep curls, elbow pull down, lat pull down, squat, and cable pull down, 3 times per week. All subjects were required to perform each repetition in a slow, controlled manner, with a rest of 2-3 minutes between sets. Training weight load was increased by 20% (3 sets of 12 repetition) at the beginning of 5^th^ week for both groups.

### Muscle biopsy

Muscle biopsy was conducted under local anesthesia (2% lidocaine) using a 18-G Temno disposable cutting needle (Cardinal Health, McGaw Park, Illinois, USA) inserted into the vastus lateralis positioned at 3 cm depth, ~20 centimeter proximal to kneecap. To prevent the potential effect of trauma caused by needle biopsy on next muscle tissue collection, the first muscle biopsy (baseline) on the vastus lateralis of right leg was conducted 3 weeks before exercise challenge. Two additional muscle biopsies were consecutively conducted in the same participants immediately after (0 h) and 48 h after the challenge for each trial. The muscle biopsy after resistance exercise (0 h) was always taken on the same location of left leg. The next muscle biopsy (48 h) was taken again on the right leg at the same distance from kneecap. Muscle tissue was quickly removed from the needle, cleaned of excess blood (when rarely needed), and disposed immediately into a conical vial containing 10% formalin. Paraffin-embedded tissue was sectioned no later than 3 h following muscle sample collection.

### Blood leukocyte

Blood samples were collected for leukocyte analysis. The total numbers of leukocyte were quantitated using an automated hematology analyzer (Sysmex XT-2000, Sysmex Corp., Kobe, Japan) according to manufacturer's instructions.

### Immunohistochemistry and Hematoxylin and Eosin (HE) staining

Double-staining immunohistochemistry on serial sections was conducted to detect p16Ink4a and CD34 in muscle tissue. A certified pathologist in Taipei Institute of Pathology, Taipei, Taiwan, conducted all histology (HE staining) and immunohistochemistry staining analyses. HE stain was used to determine leukocyte infiltration of muscle tissue. Intramyofiber and interstitial leukocytes were counted. The number of leukocyte was expressed per mm^2^. Approximately 600 muscle fibers for each biopsied sample were used to generate mean values. Muscle paraffin sections (2 μm thick) were labeled using immunohistochemistry for binding of human monoclonal antibody p16^Ink4a^ (1:200, ab108349; Abcam, Cambridge, MA, USA) [23], CD34 (1:200, ab81289; Abcam, Cambridge, MA, USA), CD163 (1:400, ab87099; Cambridge, MA, USA), CD68 (1:100, ab955; Abcam, Cambridge, MA, USA). Paraffin-embedded tissue sections on poly-1-lysine-coated slides were deparaffinized and rinsed with 10 mM Tris-HCl (pH 7.4) and 150 mM sodium chloride. Peroxidase was quenched with methanol and 3% hydrogen peroxide and slides were then placed in 10 mM citrate buffer (pH 6.0) at 100 °C for 20 min. After incubation for 1 h at room temperature, slides were washed 3 times with phosphate-buffered saline (PBS). Reactions were performed on an automated Ventana Benchmark ULTRA immunostainer, using an UltraView Universal DAB Detection Kit (Ventana, reference 760–500, Mannheim, Germany), according to the manufacturer’s instructions. The slides were then counterstained with hematoxylin. At last, the slides were photographed with the BX50 Olympus microscope (Tokyo, Japan). Negative controls were obtained by performing all of the immunohistochemistry steps, but leaving out the primary antibody. Approximately 600 muscle fibers for each biopsied sample were used to generate mean values.

### DEXA

To determine muscle mass, participants were scanned in a supine position before and after 12-week of training. Muscle mass (bone-free lean mass) was measured by a whole-body DEXA scanner (Lunar iDXA; GE Medical Systems, Madison, Wisconsin, USA) using Encore software V.13.60.033 (Encore, Madison, Wisconsin, USA) in normal mode for estimation of whole-body bone free and fat free lean body mass. For each individual, pre-testing and post-testing were conducted at the same time of the day and the participants were instructed to fast overnight and to visit the toilet prior to the scan.

### Statistical analysis

For the first study, two-way analysis of variance with repeated measure (time and group effects) was used to compare the main effect and interactive effect. Pair t test was used for pairwise comparison. A total of 6 participants would have been required for 80% power. For the second study, pair t-test was used to compare muscle mass data before and after training. Statistical significance was set at P < 0.05 for all analyses. All values are expressed as mean ± standard error (SE).
