# Correction for: Aged cells in human skeletal muscle after resistance exercise

**DOI:** 10.18632/aging.102338

**Published:** 2019-09-24

**Authors:** Chi Yang, Ying Jiao, Bing Wei, Zeyi Yang, Jin-Fu Wu, Jorgen Jensen, Wei-Horng Jean, Chih-Yang Huang, Chia-Hua Kuo

**Affiliations:** 1 Laboratory of Exercise Biochemistry, University of Taipei, Taipei, Taiwan; 2 Competitor Institute of Sports Nutrition, Beijing, China; 3 Department of Physical Performance, Norwegian School of Sport Science, Oslo, Norway; 4 Department of Anesthesiology, Far East Memorial Hospital, New Taipei, Taiwan; 5 Graduate Institute of Basic Medicine, China Medical University, Taichung, Taiwan

**Keywords:** correction

**This article has been corrected:** The authors made a mistake in size of scale bar in captions of Figures 2-5. The authors declare that this correction does not change the results or conclusions of this paper. The authors sincerely apologize for this error. The correct Figure captions are provided below. The correct scale bar size is marked in bold font.

**Figure 2. Senescent endothelial progenitor cells (p16^Ink4a+^/CD34^+^) in human skeletal muscle after resistance exercise.** (**A**) Representative immunohistochemical co-staining of muscle cross-sections (senescent cells indicated by arrows). Scale bar **55 μm.** (**B**) Senescent endothelial progenitor cells decreased in human skeletal muscle after a single bout of resistance exercise, and to a greater extends under low protein supplemented condition. * Significant difference against Baseline, P < 0.05; † Significant difference against Low Protein, P < 0.05. Low protein: 14% protein; High protein: 44% protein in weight.

**Figure 3. Phagocytic macrophage (CD68^+^) in human skeletal muscle after resistance exercise.** (**A**) Representative immuno-histochemical staining of a muscle cross-section (CD68^+^ macrophage indicated by an arrow). Scale bar **100 μm.** (**B**) Low protein supplementation before and after resistance exercise enhanced CD68^+^ macrophage infiltration in skeletal muscle above High protein trial. * Significant difference against Baseline, P < 0.05; † Significant difference against Low Protein, P < 0.05. Low protein: 14% protein; High protein: 44% protein in weight.

**Figure 4. Regenerative macrophage (CD163^+^) in human skeletal muscle after resistance exercise.** (**A**) Representative immunohistochemical staining of a muscle cross-section (CD163^+^ macrophage indicated by an arrow). Scale bar **100 μm.** (**B**) High protein supplementation before and after resistance exercise increased CD163^+^ macrophage presence in human skeletal muscle 48 h after exercise. * Significant difference against Baseline, P < 0.05; † Significant difference against Low Protein, P < 0.05. Low protein: 14% protein; High protein: 44% protein in weight.

**Figure 5. Centrally nucleated fibers in human skeletal muscle after resistance exercise.** (**A**) Representative hematoxylin and eosin staining of a muscle cross-section (centrally nucleated fibers indicated by arrows). Scale bar **100 μm.** (**B**) (**††**) No difference between Low and High protein trials was found. *Significant difference against Baseline, P < 0.05. Low protein: 14% protein; High protein: 44% protein in weight.

Original article: Aging. 2018; 10:1356–1365. 1356-1365 . https://doi.org/10.18632/aging.101472

